# Editorial: Gut microbiome-driven pathogenesis and intervention strategies in liver diseases

**DOI:** 10.3389/fmed.2026.1973834

**Published:** 2026-10-06

**Authors:** Ze Xiang, Yijian Chen, Renlei Ji, Xuyong Wei, Xiao Xu

**Affiliations:** 1 Zhejiang University School of Medicine, Hangzhou, China; 2 Department of Hepatobiliary and Pancreatic Surgery, Affiliated Hangzhou First People’s Hospital, School of Medicine, Westlake University, Hangzhou, China; 3 The Second School of Clinical Medicine, Zhejiang Chinese Medical University, Hangzhou, China; 4 Department of Cancer Biology, Dana-Farber Cancer Institute, Boston, MA, United States; 5 Department of Biological Chemistry and Molecular Pharmacology, Harvard Medical School, Boston, MA, United States; 6 Hepatobiliary Center and Transplantation Center, The First Affiliated Hospital with Nanjing Medical University, Nanjing, China

**Keywords:** dysbiosis, gut microbiome, gut-liver axis, liver disease, microbial metabolites, microbiome-based therapy

The gut and liver are closely linked through the portal circulation and biliary system. This relationship has long been recognized, but microbiome research has given it new biological and clinical meaning. Gut-derived microbial products and metabolites reach the liver directly, while bile acids and other hepatic secretions help shape the intestinal environment. Disturbance at either end can therefore affect the entire gut-liver axis (1).

The term “dysbiosis,” however, should be used with care. A change in bacterial abundance does not by itself explain liver injury. What matters is microbial function: which metabolites are produced, whether the intestinal barrier remains intact, and how the host responds. Increased exposure to microbial components, together with changes in bile acids, short-chain fatty acids, tryptophan metabolites, ammonia, and endogenous ethanol, may influence hepatic metabolism, inflammation, fibrosis, and immune surveillance (2).

Similar microbial alterations have been reported in metabolic dysfunction-associated steatotic liver disease, alcohol-associated liver disease, viral hepatitis, cirrhosis, and hepatocellular carcinoma. This does not mean that these diseases share a single microbial cause. Diet, geography, age, medications, and disease severity all affect the microbiome. In advanced liver disease, dysbiosis may also result from altered bile acid metabolism, intestinal motility, malnutrition, or antibiotic exposure. Most human studies remain cross-sectional, making it difficult to distinguish cause from consequence.

The clinical value of microbiome research will depend on whether these observations can be reproduced and used in practice. Differences in sample Research Topic, sequencing, and bioinformatic analysis still limit comparisons between studies (3, 4). Candidate microbial biomarkers should be validated in independent populations and tested against established clinical tools. A signature that separates patients from healthy controls within one dataset is not necessarily useful for diagnosis or prognosis.

Microbiome-directed treatments are equally promising and uncertain. Diet, probiotics, prebiotics, synbiotics, antibiotics, fecal microbiota transplantation, bacteriophages, and engineered microorganisms may modify parts of the gut-liver axis (5). Yet a change in microbial composition is not the same as clinical benefit. Treatment effects are likely to depend on the underlying disease, disease stage, baseline microbiome, medication use, and host immune status. Safety is especially important in patients with cirrhosis, who may have impaired barrier function and increased susceptibility to infection.

The field has moved beyond asking whether the gut microbiome is associated with liver disease. The more difficult questions are when these changes matter, whether they are causal, and whether they can be modified safely. Progress will require disease-specific mechanisms, reproducible methods, and trials with clinically meaningful endpoints. The microbiome is likely to become part of hepatology, but it should not be treated as a universal explanation or a universal treatment.
